# Toward Mass Video Data Analysis: Interactive and Immersive 4D Scene Reconstruction

**DOI:** 10.3390/s20185426

**Published:** 2020-09-22

**Authors:** Matthias Kraus, Thomas Pollok, Matthias Miller, Timon Kilian, Tobias Moritz, Daniel Schweitzer, Jürgen Beyerer, Daniel Keim, Chengchao Qu, Wolfgang Jentner

**Affiliations:** 1 Department of Computer and Information Science, Universiät Konstanz, Universitätsstr. 10, 78465 Konstanz, Germany; matthias.miller@uni.kn (M.M.); timon.kilian@uni.kn (T.K.); daniel.schweitzer@uni.kn (D.S.); keim@uni.kn (D.K.); wolfgang.jentner@uni.kn (W.J.); 2 Fraunhofer IOSB, Fraunhoferstr. 1, 76131 Karlsruhe, Germany; thomas.pollok@iosb.fraunhofer.de (T.P.); tobias.moritz@iosb.fraunhofer.de (T.M.); juergen.beyerer@iosb.fraunhofer.de (J.B.); chengchao.qu@gmail.com (C.Q.); 3 Vision and Fusion Lab (IES), Karlsruhe Institute of Technology (KIT), c/o Technologiefabrik, Haid-und-Neu-Str. 7, 76131 Karlsruhe, Germany

**Keywords:** 4D reconstruction, visual exploration, computer vision, machine learning, forensics, virtual reality, surveillance systems

## Abstract

The technical progress in the last decades makes photo and video recording devices omnipresent. This change has a significant impact, among others, on police work. It is no longer unusual that a myriad of digital data accumulates after a criminal act, which must be reviewed by criminal investigators to collect evidence or solve the crime. This paper presents the VICTORIA Interactive 4D Scene Reconstruction and Analysis Framework (“ISRA-4D” 1.0), an approach for the visual consolidation of heterogeneous video and image data in a 3D reconstruction of the corresponding environment. First, by reconstructing the environment in which the materials were created, a shared spatial context of all available materials is established. Second, all footage is spatially and temporally registered within this 3D reconstruction. Third, a visualization of the hereby created 4D reconstruction (3D scene + time) is provided, which can be analyzed interactively. Additional information on video and image content is also extracted and displayed and can be analyzed with supporting visualizations. The presented approach facilitates the process of filtering, annotating, analyzing, and getting an overview of large amounts of multimedia material. The framework is evaluated using four case studies which demonstrate its broad applicability. Furthermore, the framework allows the user to immerse themselves in the analysis by entering the scenario in virtual reality. This feature is qualitatively evaluated by means of interviews of criminal investigators and outlines potential benefits such as improved spatial understanding and the initiation of new fields of application.

## 1. Introduction

Image and video footage is becoming increasingly important for criminal investigation, as more and more sensors, from security cameras to mobile phones, are easily available and in use. This has an impact on the accumulation of data that needs to be thoroughly investigated, which is often done manually and thus time-consuming and cost-intensive. The German police expects approximately 8 h of investigation time for one hour of video material [1]. In cases where the police ask citizens to upload video or image data for an incident, it is expected that several images will be uploaded, capturing the same content from different perspectives. Famous examples are the Boston Marathon Bombing and New Year’s Eve at Cologne Cathedral [2]. Investigators determine the relevance for the data provided and note whether the supplied video records the scene of interest at the time of interest. A second step is spatial localization, which is used to determine the location of the camera sensor and its field of view. The third step is temporal localization, which establishes a temporal relationship between the other available video data. Finally, a detailed analysis of the video and image content is performed to identify objects, persons, and scenes of interest necessary for the particular case.

Our proposed visual analytics approach (ISRA-4D 1.0) supports the user in all these tasks. It automates processes to a great extent, while still allowing the user to intervene and optimize during all steps. The processed scene is composed of a 4D scene that combines multiple video sources synchronized on a single timeline. Users can explore this 4D scene in our interactive 4D scene investigator, tracking objects across various video feeds, annotating scenes, and exploring the scene in virtual reality, which significantly improves the perception of distances, angles, and details of the scene. In the long-running VICTORIA project (https://www.victoria-project.eu/), numerous internal and external stakeholders underlined the importance and necessity of such an approach for their daily work. Furthermore, additional use cases could be identified.

When massive amounts of data are available, for instance, through upload platforms asking the public to upload videos of an incident, the police is often confronted with a lot of irrelevant material. Our scene reconstruction approach can automatically determine whether specific images and videos where taken of a specific scene. Therefore, the police needs to reconstruct a static scene where the incident took place. Afterward, the reconstruction algorithms can determine whether the additionally uploaded video material fits into the scene or not. This approach is robust, as for videos multiple frames are available and thus more evidence can be gathered.

The primary use case is, however, crime scene reconstruction, where image material can be collected from witnesses in combination with image material recorded by the police after the incident. The constructed 4D scene can then be further annotated and explored using ordinary desktop computers and virtual reality with available consumer hardware. This allows persons involved in the case to better understand and orient themselves at the crime scene, even if they may not have seen it in reality. Additionally, such scenes can be digitally archived and also used in court.

Besides, the framework can be deployed for efficient monitoring of critical infrastructures and public places, such as airports, train stations, or industrial areas. The state-of-the-art uses arrays of monitors showing the live streams of cameras. Such an array of monitors is difficult to oversee, and important events can easily be overlooked. In addition, it requires a constant cognitive workload to recognize and remember position and orientation of each camera, which makes it increasingly difficult to trace moving objects. Our approach allows the embedding of cameras into the 4D scene, whereby the images can be projected into the scene in real-time. Additionally, the proposed concept can be used for mission planning and training for special forces in which virtual reality is an essential component. It allows users to spot a scene using drones, video glasses, or other imaging sources and receive a 3D scene that can be virtually inspected to plan the mission. Especially, the collaborative virtual reality and mixed interactions with desktop access that provide an overview are considered useful.

This work is a direct extension of an earlier publication [3] in which the predecessor framework is presented in less detail. In the line with this work, this publication contributes (1) a modular pipeline approach for the reconstruction of static 3D and dynamic 4D scenes, (2) a visual interface concept for the interactive and immersive exploration of such scenes, and (3) four use cases demonstrating the manifold applicability of our approach. The 4D scene reconstruction pipeline is carefully constructed to increase its robustness, extensibility, and user handling. The final reconstructed scene can be investigated on desktop computers, providing a good overview of the progression of events. Additionally, virtual reality allows the operator to immerse into the scene where distances, angles and orientation are perceived as in reality. The scene can be further annotated and investigated using various tools for spatial and temporal analysis to find interesting locations and times within the scene and timeline. Furthermore, our approach allows the operator to always intuitively access the original material that has been used to reconstruct the scene as well as the dynamic material that is blended into the scene.

## 2. Related Work

In this section, first, an overview of existing approaches and techniques is provided which can be used to create a 3D reconstruction of dynamic and static environments, as well as methods to analyze the content of videos. Subsequently, current state-of-the-art multi-video surveillance systems are presented. Finally, the use of visual and immersive analytics approaches in different domains is outlined.

### 2.1. 3D Scene Reconstruction

The reconstruction of dynamic objects or static scenes with state-of-the-art approaches like Simultaneous Localization and Mapping (SLAM) [4] or Structure from Motion (SfM) [5] is challenging. The quality of the reconstruction depends significantly on the quality of the footage. Zhong et al. presented the “Detect-SLAM” framework, which combines an object detection approach with a reconstruction technique for a more robust 3D reconstruction of dynamic objects [6]. By initially segmenting the scene material, their framework improves the reconstruction quality and detection rate even from disadvantageous viewpoints and potential occlusion. Similarly, Bullinger et al. integrated algorithms and segmentation techniques based on optical flow to compute object-specific motion cues and corresponding points [7]. The combination of SfM and triangulation enables 3D scene reconstruction and simultaneous tracking of static and dynamic entities in a scene. However, a typical limitation of such an approach is the availability of binocular footage. Besides the reconstruction of 3D objects, the extraction of a three-dimensional scene from 2D images is another challenging research area that deals with the restoration of dynamic 3D scenes. This endeavor requires elaborate routines to mitigate the negative effects of inaccuracy and uncertainty in dynamic scenarios.

Mustafa et al. presented an approach to improve an initially sparse 3D scene reconstruction using traditional reconstruction techniques with a joint optimization framework [8]. Their approach is applicable to scenarios with moving cameras without prior knowledge of the scene structure, whereas earlier techniques were often limited to fixed camera positions in the scene. They take into account data, contrast, smoothness, and temporal characteristics to narrow the solution space and achieve a clean depth restoration for multiple synchronized and unsynchronized input videos. In contrast, Ji et al. presented a method for the 3D reconstruction of dynamic scene objects based on video synchronization that exploits locally rigid patches without the need for segmentation [9]. However, these approaches are limited to a few large, moving foreground objects in a scene. Therefore, they cannot always be applied to real-world scenarios that might contain diverse, dynamic objects, such as crowds of people or cars.

In the current work, especially for the reconstruction of dynamic scenes, several different approaches are realized and compared. The most visually appealing approach was selected and implemented as a module of the preprocessing pipeline.

### 2.2. Video Content Synthesis: Object Detection and Re-Identification

The generation of data about image content, such as the detection of objects in images and videos, is a common task used in numerous domains. One area that receives a lot of attention is research on real-time object detection. For example, YOLO, introduced by Redmon et al., is a framework based on neural networks enabling the detection of objects within images with little computational effort [10]. In later years, gradual improvements of the YOLO framework were presented: YOLOv2 [11] and YOLOv3 [12]. Besides, many alternative approaches for real-time object detection in videos were established, such as SSD [13] and R-FCN [14].

In addition to the mere object detection within an image, it is also essential to identify the same object during a video or in different footage. The so-called object re-identification task is a very challenging and error-prone task, e.g., due to context-related problems like occlusion, noise, varying illumination, moving background objects, and ambiguity [15,16]. Li and Loy presented an approach that allows the re-identification of objects in successive frames, even if an object could not be identified in the frames in between [17]. Their segmentation-based approach allows visual tracking of objects that even change in scale and rotation. While this approach focuses on object re-identification in a single camera, others specialize in object tracking through multi-camera systems. For example, Bialkowski et al. [18] presented a database for the re-identification of persons with videos that record the same environment from different angles and under different lighting conditions. They demonstrated the dataset using a simple re-identification system that compares detected objects between different cameras. The presented approach requires overlapping viewports of the cameras. Other approaches are even more sophisticated and support the tracking of objects through non-overlapping camera networks [19,20]. Beyond the extraction of movement trajectories, several approaches aim to analyze the movement of detected persons further. For example, Devanne et al. analyzed the trajectories of skeletons and focused on the recognition and classification of actions within the movement of a person [21]. Goffredo et al. dealt with gait analysis in surveillance videos [22]. The way a person walks is very individual, making it possible to use gait characteristics for person re-identification.

Depending on the choice of the object detection and re-identification approach, the run times and results vary. Thanks to the modular design of the current approach, new improvements of such models can easily be implemented in the pipeline. In the current version of the presented framework, a pretrained YOLO v3 module [12] was used in combination with a state-of-the-art re-identification approach.

### 2.3. Multi-Video Surveillance Systems

Another research focus is on the optimization of multi-camera surveillance systems. Here, the dominant goal is to contextualize heterogeneous video sources with different viewports, light and color differences, and structurally different parameters (e.g., camera intrinsics). For example, Collins et al. presented a framework for the seamless tracking of moving objects through a network of surveillance cameras [23]. A site model of the monitored environment and calibrated cameras are required to calculate the trajectories of objects. There are alternative approaches that do not require a spatial model of the environment with calibrated cameras, but rather estimate relative camera locations and their intrinsic parameters on the fly. For example, Javed et al. presented a large-scale surveillance system that automatically calculates the spatial relation between the cameras [24]. The system detects and tracks objects and persons across multiple cameras. First, the tracks of objects are computed for each camera. Then, a match between the views of the same object by multiple cameras is calculated. This makes it possible to find relationships between the field of view lines of different cameras without explicit camera calibration. Several approaches in literature (see, e.g., in [25,26]) follow a similar principle for scenarios where it cannot be assumed that there is sufficient visual overlap occurs which would allow a purely visual camera correspondence estimation.

Other work deals with the quantification of camera constellations, calibrations, and image content. For instance, Zaho and Cheung presented a technique for optimizing the camera placement in a multi-camera system by measuring and comparing the performance of different camera constellations for object and face detection tasks [27]. Lim et al. suggested an approach for automatic, image-based calibration of stationary cameras [28], i.e., the automatic configuration of pan, zoom, and tilt parameters of cameras in multi-camera systems to optimize the system’s overall performance. Beyond that, Shen et al. proposed an approach to quantify the content of surveillance cameras to prioritize the views of specific cameras in multi-camera surveillance systems [29].

The current framework comprises publicly available, state-of-the-art approaches for object detection and re-identification. The output of these models is used to improve dynamic point cloud generation processes and to simultaneously display high-level information from multiple videos in a shared 3D environment. With the modular design of the introduced framework, it is possible to adapt to further advances in this area by exchanging individual modules in the preprocessing pipeline and adapting their output to the required format.

### 2.4. Visual and Immersive Analytics

Visual analytics has proven to be a valuable tool for explorative and confirmatory analysis tasks [30,31,32,33]. With the help of visualizations, hidden information in the data can be spotted without a concrete definition of a hypothesis. In contrast to merely statistical evaluations, this makes it possible to keep users up to date during interactive data analysis procedures. Various visual analytics solutions have also been developed in the field of police and law enforcement. For example, Malik et al. presented an instrument for police resource allocation and predictive analytics [34]. Various other works also deal with the identification of criminal hotspots and use visual analytics procedures to facilitate the process [35,36]. Sacha et al. introduced a tool for the interactive analysis of spatio-temporal metadata of crime reports using abstract data visualizations such as correlation matrices and scatterplots [37]. Similarly, Jentner et al. analyzed crime reports, but focused on the analysis of patterns to provide insights on a large bulk of data and to find clusters of similar crimes [38].

Virtual reality has been frequently used for simulation [39], training [40], and educational [41] purposes due to its ability to immerse users in virtual environments. Virtual content can be observed more naturally, conveying the impression to experience a real situation. Immersive analytics [42] is a relatively new field in which visual analysis procedures are performed in immersive environments such as augmented or virtual reality environments. Previous research has identified several benefits associated with induced immersion. For example, Probst et al. used VR to explore large chemical spaces in which molecules are depicted as volume visualizations [43]. They concluded that VR provides a more intuitive exploration process, which is particularly useful for educational and training purposes. Zhang et al. found a benefit of VR in terms of understanding geometric structures in VR and attribute this effect to the natural inspection of 3D objects, which is similar to the inspection of physical objects in the real world [44]. Similar effects were also reported regarding more abstract data visualizations. For instance, Donalek et al. reported a better perception of the datascape geometry in graph visualizations when participants were immersed in VR [45]. Further benefits have been identified in terms of data validation [46], collaboration [45], increased task performance on specific data exploration tasks [47,48], and memorability [49]. Etemadpour et al. found that especially surface-based visual encodings profited from a stereoscopic perception in VR [50].

The use of visualizations for the analysis and extraction of knowledge from data has proven itself in the past. Therefore, visualizations are used in the current framework to facilitate the analysis process of mass video data. Recent developments in immersive analytics research could demonstrate various advantages of using virtual reality in the visualization context. The current framework allows users to observe 4D scene reconstructions in VR in order to exploit these benefits.

## 3. Crime Scene Analysis Framework: Processing Pipeline

**Figure 1:**
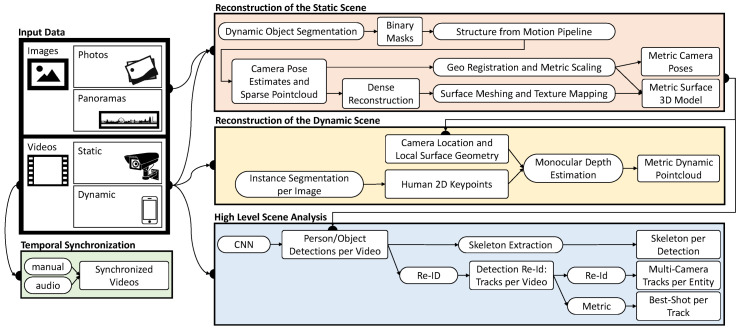
Processing pipeline of the crime scene analysis framework. Multimedia input data are processed in three main steps: First, a static reconstruction of the crime scene is created using a structure-from-motion approach. Second, dynamic elements are extracted as dynamic point clouds. Third, tracks of persons and objects are extracted using machine learning models.

In order to explore heterogeneous data sources in a shared 3D reconstruction, the underlying data needs to be preprocessed. In this section, a detailed overview of the used preprocessing pipeline is provided (see Figure 1). First, all supported input data types and data-specific terms are introduced. Subsequently, the approaches used for static and dynamic scene reconstructions as well as metadata extraction (high-level scene analysis) are explained. The section concludes with the description of the module for temporal synchronization.

### 3.1. Input Data

The crime scene analysis framework presented is optimized for the rapid analysis of large amounts of image and video data from a certain incident. For example, after a shooting in a city center, sources could be recordings from surveillance cameras as well as photos and videos taken by eyewitnesses with their mobile phones. Therefore, the resulting set of data sources can be very unstructured and difficult to analyze. The two main sources are static cameras that do not move and maintain their perspective (static camera), and moving cameras that record different locations throughout the incident (dynamic camera). To further enhance the context, image and video material from the time before or after an incident can also be integrated into the framework by registering it solely in space, without considering time. These sources are *time-independent*, as they are not registered to a certain point in time of the progression of events to be analyzed.

Besides video data, image data such as individual photos, panoramas, and photo spheres can also included in the analysis. By default, such footage is currently treated as time-independent and is only registered in space. This could, for example, comprise images and photo spheres taken from the place of interest after the incident in the forensic analysis and help investigators to compare the environment at the time of the incident with the environment shortly after the incident.

### 3.2. Reconstruction of the Static Scene

The 4D reconstruction pipeline is divided into a static scene reconstruction and a dynamic scene reconstruction. In the latter, all dynamic objects are reconstructed for each frame and mapped into the previously created static surface reconstruction (see Section 3.3). In order to support the investigator in navigating through a large multimedia database of an event, our approach first reconstructs the static scene. The goal is to provide the user with a 3D surface model and a cadastre of all cameras in the scene. This approach spatially structures the data and provides the user with a big picture of the scene, which means that a scene no longer needs to be investigated on basis of video files. This enables investigators to directly understand the geometric relationships of camera locations and their viewing direction as well as objects within the scene.

As depicted in Figure 1, the static scene reconstruction pipeline starts with a dynamic object segmentation step. Moving objects like cars or persons can lead to inconsistent 3D reconstructions because feature points of these objects change their 3D position over time. Therefore, binary masks are created for each image to indicate whether a pixel is static or not. MaskRCNN is a neural network that creates such instance boundary segmentations for individual objects in the scene such as persons, cars, or bicycles [51]. For this task, we used a publicly available pretrained model that was trained using the mscoco dataset [52]. All other classes are considered static. It is important to note that these binary masks may not always be perfect, for example, a person displayed on an advertising board is actually a static image, but is still identified as a person by the network. However, the masks are only used to filter out areas that most likely contain dynamic content to allow for a high-quality static reconstruction.

Subsequently, the locations of all cameras and a sparse static point cloud are restored using a Structure-from-Motion (SfM) approach. Previously calculated masks are used to exclude dynamic elements in images from this procedure. The current approach integrates COLMAP, a state-of-the-art SfM pipeline [5]. SfM is a technique which first attempts to identify correspondences between all images by means of image feature point detection and matching, such as SIFT [5]. From the set of all possible 2D-2D correspondences, the camera locations as well as a sparse point cloud of valid correspondences are restored and globally optimized using Bundle Adjustment (BA) [53]. Cameras that could not be spatially registered can be manually positioned.

**Figure 2:**
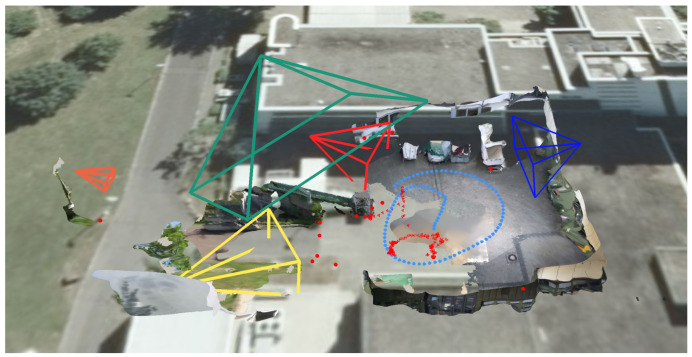
3D reconstruction after being manually geo-registered into satellite imagery based map data.

Once the sparse reconstruction is completed, a dense reconstruction is started. In this phase, a multi-view stereo (MVS) reconstruction is performed by employing OpenMVS, resulting in a textured surface model of the scene [54]. The surface model and camera locations resulting from the SfM and MVS pipeline are usually not metrically scaled or georegistered. However, in order to be able to measure the distances between individual points in the scene or to obtain the geolocation of a selected 3D point, absolute scaling or georegistration must be performed (see Figure 2).

In case GPS metadata is available, georegistration can be performed automatically as long as there is sufficient valid GPS metadata. When the respective options are enabled, images from smartphones usually contain a geo-tag. However, videos usually do not contain GPS metadata, unless they were captured by specialized drones that create separate GPS logs. The reason for that is that it would require a GPS tag per frame which does not fit in the standardized EXIF metadata field. Therefore, the prototype contains a manual georegistration approach, which enables users to manually align the reconstructed scene using a satellite map with elevation data on a 3D globe, similar to Google Maps. This makes it possible to add several separate, non-overlapping reconstructions which may be relevant to a case, but are physically located at different locations. In case map data is not available due to indoor footage, the reconstruction has to be scaled metrically by specifying a measure of a known object like the height of a door or the size of a tile on the floor. However, geo-information is still missing and one can only interact with the model in a local metrically scaled coordinate space.

### 3.3. Reconstruction of the Dynamic Scene

#### 3.3.1. Classical Stereo Depth Estimation

**Figure 3:**
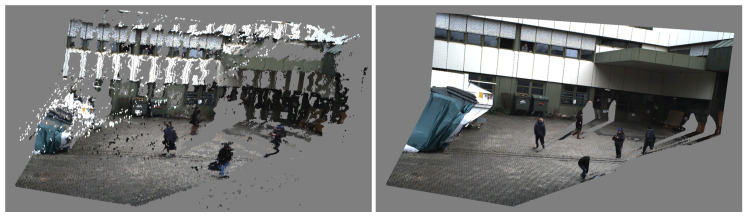
(**Left**) Point cloud reconstructed from a stereo camera using classical stereo block matching. (**Right**) Point cloud reconstructed with our geometrically based monocular depth reconstruction.

The reconstruction of a dynamic scene, i.e., a sequence of video frames with dynamic content, is a challenging task. In the past, point clouds were typically reconstructed from calibrated and synchronized stereo image pairs or multi-view setups. Disparity maps were computed by exploiting the epipolar stereo geometry. There are different block matching approaches that assign each pixel of the left image to the best matching pixel of the right image along its epipolar line by comparing local image blocks with correlation-based block matching approaches [55,56]. These classical approaches allow an efficient reconstruction of any image structure without having to rely on high-level information of scene content. An example result of a point cloud that has been reconstructed using stereo block matching is shown on the left in Figure 3. The drawback, however, is its inherent dependency on synchronized image pairs from at least two different cameras that overlap. In practice, stereo cameras are not available in most video security applications and cameras are preferably installed in different viewing directions to reduce the total number of cameras and thus hardware costs.

#### 3.3.2. Neural Network-Based Monocular Depth Estimation

**Figure 4:**
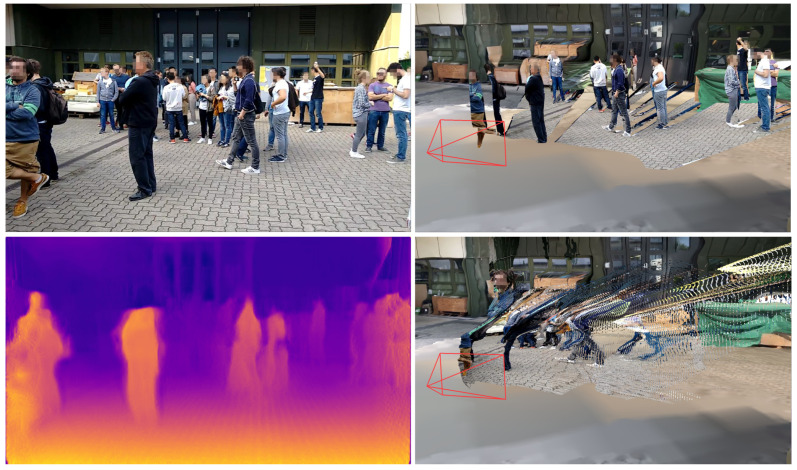
(**Top left**) Input image. (**Top right**) Result of our method, in which people are segmented and placed upright on the ground.(**Bottom left**) Resulting depth map using Monodepth2. (**Bottom right**) Embedded point cloud generated using Monodepth2.

Different approaches are worth to be considered to deal with non-synchronized heterogeneous monocular image data. We focused on approaches that are frame-based and capable of obtaining a depth map, point cloud, or other high-level representation for each frame independently of other frames. This enables high parallelization and thus fast processing. In recent years, monocular depth estimation using neural networks grew in popularity. Several methods like Monodepth2 [57], monoResMatch [58], and a self-monitored monocular depth estimation approach by Hermann [59] were considered. An exemplary result of Monodepth2 with a pretrained model based on the KITTI dataset [60] is shown in the bottom line of Figure 4. While the results of these approaches look impressive from a scientific point of view, these point clouds did not meet the requirements for the application, as they were still too noisy or stretched. These artifacts become particularly visible when the perspective of the observing camera on the point cloud is changed. Even the slightest variation in depth on the surface of a human body makes it difficult to identify the person in the scene. This does not mean, however, that these approaches are not suitable in general. Recent work that explicitly dealt with monocular depth estimation, for instance, with focus on videos with humans [61] or for obstacle detection in autonomous cars [62], showed that monocular depth estimation from neural networks could be a promising technique in the future.

#### 3.3.3. Object Detector-Based Dynamic Object Placement

**Figure 5:**
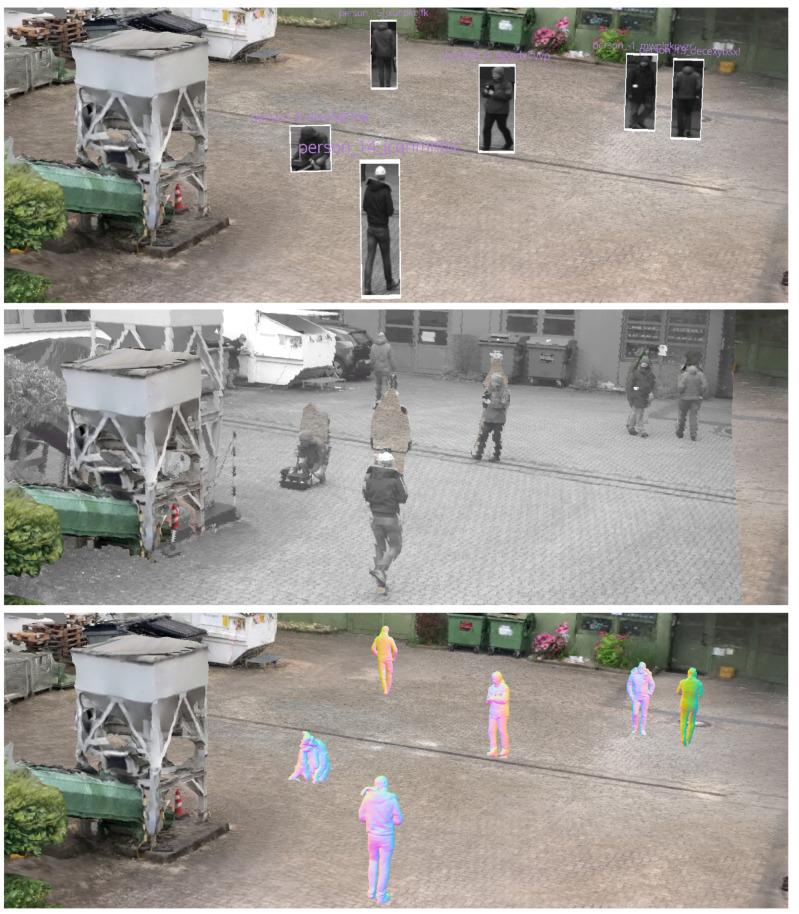
Dynamic objects can be displayed differently in the static 3D reconstruction. (**Top**) Detected bounding boxes of persons are embedded upright. (**Center**) Complete depth map of the segmented image is superimposed. (**Bottom**) People reconstructed with PIFuHD are embedded.

To avoid the previously mentioned noisy artifacts in the final scene, it is possible to focus only on detected dynamic elements in the scene. This monocular reconstruction approach is limited to detected objects (e.g., persons and cars) and positions them in the already reconstructed static scene geometry. A generic object bounding box detector can be used for the detection of multiple classes such as pedestrians, bicycles, cars, and trucks. The 3D object location of each object can be calculated by intersecting the bottom edge of the bounding box with the surface mesh of the static scene reconstruction. The image patches of the detected objects are then placed individually in the scene, similar to billboards, so that the objects are upright. A result of such an embedded dynamic reconstruction is shown at the top of Figure 5.

#### 3.3.4. Orthogonal Depth Estimation Approach

**Figure 6:**
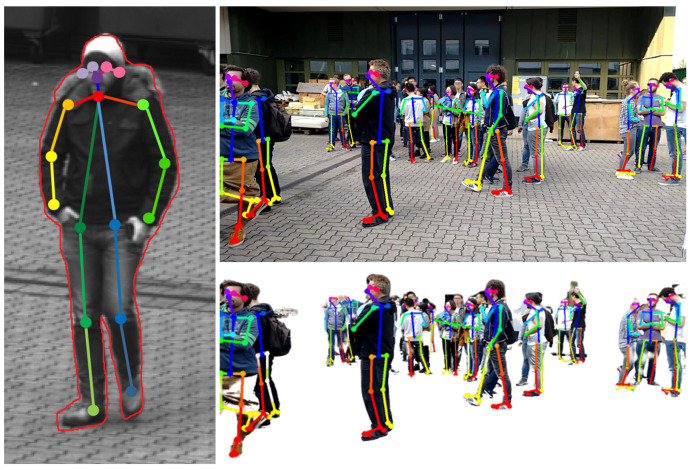
(**Left**) OpenPose annotation key points. The red silhouette represents the segmented instance boundary when using MaskRCNN. (**Top right**) Exemplary OpenPose result on an image with several persons and partial occlusions. (**Bottom right**) Neural network-based automatic foreground segmentation of people. This foreground is the dynamic part of the image that has to be placed in the scene as dynamic content.

In the current framework, an orthogonal depth estimation approach is used (see Figure 1) as an improvement of the previously presented approach with bounding box detectors. The proposed prototype integrates MaskRCNN, an instance segmentation approach based on neural networks that can segment individual objects [51]. As shown in Figure 6 (left) as a red silhouette, the segmentation is capable of cutting out objects in more detail. Similar to the previous approach, the 3D location of segments is then calculated with the aid of the underlying static 3D reconstruction. The resulting depth map can be superimposed over the static mesh, preserving all pixels of the original input image (see Figure 5, center).

A skeleton extraction module increases the stability of the procedure for detected persons. The neural network-based approach OpenPose allows to obtain 2D key point locations of a person [63]. These key points include body parts such as feet, shoulders, elbows, hands, head, nose, and ears, as shown in Figure 6. An advantage of this approach is that it is possible to obtain high-level information about a person’s body, even if the person is partly occluded, e.g., by another person or object. A person’s skeleton information is used to improve the depth estimation for the corresponding image segment. The framework assumes that a person is on average 1.70 meters tall and that the feet always touch the ground. Handstands, jumps, or other artistic postures are currently not supported by our approach. However, in most cases of visual surveillance this is not a major limitation. The 3D location of a person is calculated by casting a ray that intersects the pixel position of the foot with the 3D surface model, provided that the camera position and the intrinsic camera parameters are known. In case the location of the foot could not be restored, other known key point locations are used and an estimated offset is added. Thus, if the segmentation algorithm only returns, for example, the upper body of a person due to occlusion, the extracted skeleton key points indicate this circumstance. The 3D location of the segment is then calculated taking into account that the entire person is actually larger than the extracted segment, resulting in a more accurate 3D position.

#### 3.3.5. Neural Network-Based Full Body Reconstruction

**Figure 7:**
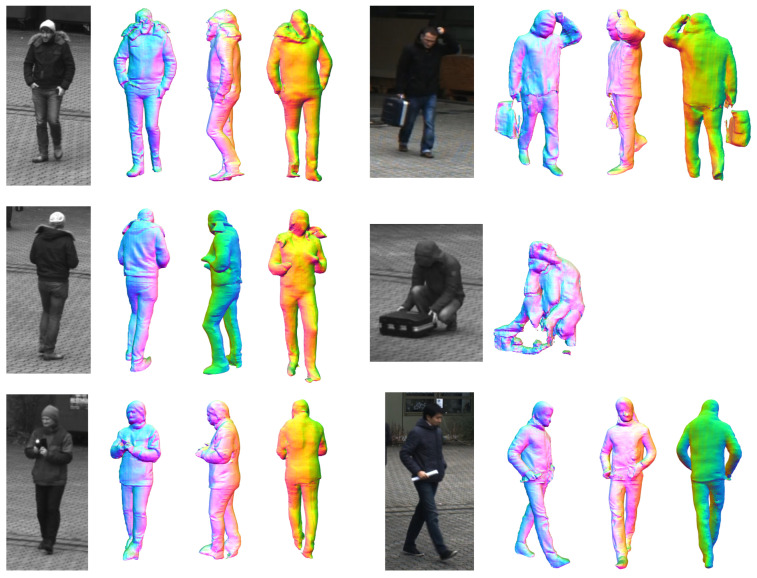
3D models of different persons reconstructed from single images using PiFuHD. The reconstruction time was approximately 10 seconds per person. The size of the image patches varied between 260 × 330 and 440 × 960 pixels. Most models were successfully reconstructed from all sides. Only the kneeing man opening a suitcase (lowest resolution) could not be reconstructed from the back.

Recent developments in artificial intelligence also enable the reconstruction of the complete 3D body shape of a clothed person from monocular image data [64]. This means that even if only the front of a person is visible, the back can be estimated from the network. The results of this approach are shown in Figure 7. In addition to the reconstruction of the body shape, the entire body texture can be reconstructed from a single monocular image or even with multiple views [65]. By reconstructing each person individually for each frame, these models can be inserted into the scene and give the impression of actually animated people walking through the scene (see Figure 5, bottom). In the scope of this work, we experimented with a pretrained model provided by the PIFuHD authors that was trained using a synthetic dataset.

The different approaches described in this section can be selected according to the needs of users. In some cases, where the user prefers to rely on fast processing methods such as the display of image snippets in the static scene, fast analysis capabilities are especially important. In other cases, a more detailed and accurate 4D reconstruction is important. While some approaches retain all information from the input material at the price of artifact-afflicted representations (e.g., superimposition of the entire depth map), others rely on object detection algorithms and may withheld information during the visual exploration (e.g., 3D models of persons). The choice of the most appropriate approach is always a compromise and must be considered for each individual application.

### 3.4. High-Level Scene Analysis

**Figure 8:**
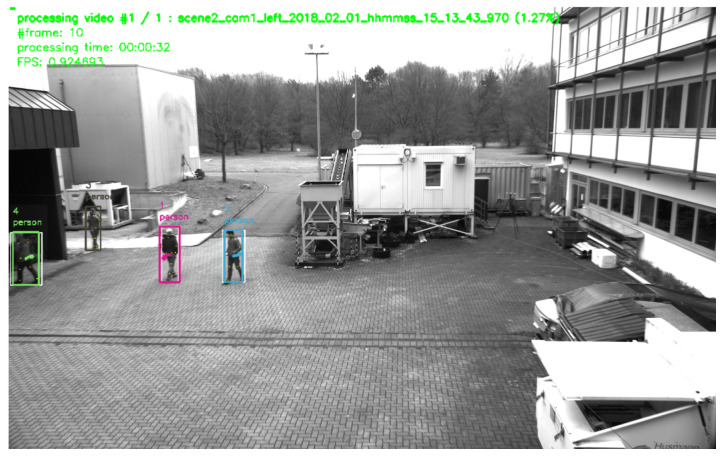
Frame taken from feature detection preprocessing procedure. During processing, the original video is played back while detected objects are highlighted.

After starting the main application, the system checks whether feature preprocessing has been performed beforehand. If not, the preprocessing sequence will be started and each *time-dependent* video is processed in an object detection pipeline. The pipeline for the high level scene analysis (see Figure 1) has a modular structure. This way, the entire pipeline or parts of it can be replaced by other modules that deliver an output using the same format. Figure 8 shows a frame from the feature extraction preprocessing step. During preprocessing, the video is played back and all recognized objects and persons are highlighted by colored rectangles, including their respective path of movement.

#### 3.4.1. Object Processing in Camera Space

Most of the feature extraction pipeline takes place in camera space. The position of detected objects is described in pixel coordinates, based on the frame in which they were detected. All further steps of feature processing (e.g., skeleton extraction and re-identification) are image feature-based and therefore do not make use of the position itself. In a subsequent step, which is described in the next paragraph, these pixel coordinates are mapped to world coordinates using the estimated camera pose from the reconstruction.

**Figure 9:**
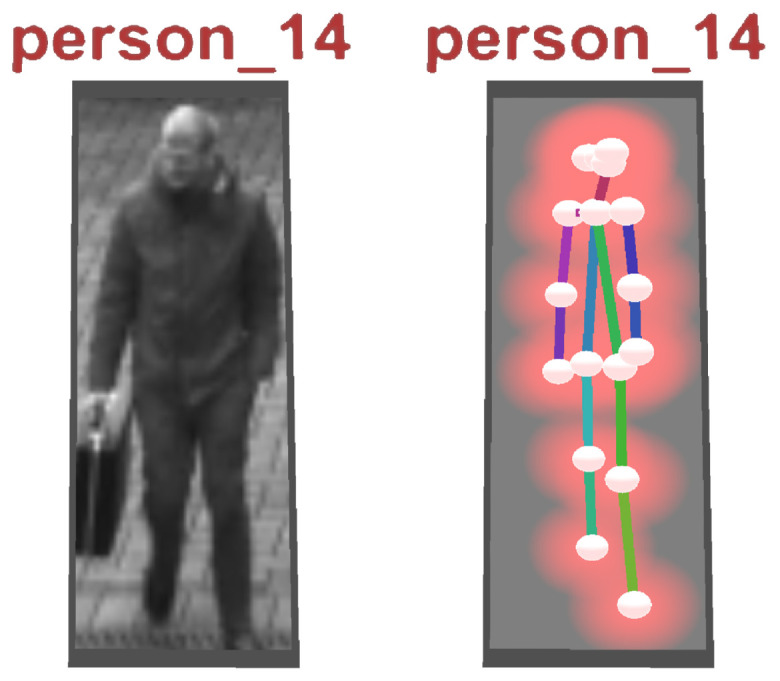
For each person recognized in a video frame (**Left**), OpenPose is applied for skeleton extraction. The extracted skeletons can later be displayed in the scene as connected points (**right**).

As a first step in the feature extraction pipeline, each video from the input pool is processed in an object detection module (convolutional neural network (CNN)) that extracts all detected entities (persons and objects) for each frame of the video. In the present case, the YOLO v3 library (https://pjreddie.com/darknet/yolo/) is applied. The network was used pretrained on the mscoco dataset [52]. The result is a set of independent *Detections* for each frame, each containing information about its location in the image space (bounding box), a confidence score, and a classification of the object type (e.g., car, person, and backpack). In a second feature extraction step, each detection classified as “person” is processed in a skeleton extraction module by using the mentioned bounding box coordinates of the detection as input parameter. The current status of the proposed framework includes the OpenPose skeleton extraction library (https://github.com/CMU-Perceptual-Computing-Lab/openpose). Similar to the YOLO module, the OpenPose network was used pretrained on the mscoco dataset [52]. With that, each detection of the class “person” is enriched with skeleton information representing the key points of the detected skeleton. Figure 9 depicts a skeleton (right) as shown later in the exploration framework for the detection of a person (left). Subsequently, for each video, all detections are processed in a re-identification module, comparing the detections from different frames and identifying all detections that belong to the same entity (*Track*). The current implementation exploits DeepSort (https://github.com/Qidian213/deep_sort_yolov3), which reuses features from the object detection module (YOLO v3). As a result, a set of tracks is available for each video, each of which containing detections from the same entity (e.g., person and car) that describes its spatial movement over time. For each track, a representative detection is selected in which the entity is optimally represented (*Best Shot*). Currently, the detection with the highest confidence (YOLO output) is selected as best shot. Last, a global re-identification module is used to find relations between tracks of different videos. The detection features from the object detection module are used to compare the tracks of different videos and form sets of tracks that belong to the same entity.

#### 3.4.2. Position Mapping to World Space

After each video is passed through the object detection pipeline, the position information of the detections remains in camera space (frame coordinates in the pixel space). To locate the detection positions in the 3D scene, the corresponding 3D coordinates are computed based on the extrinsic and intrinsic parameters of the respective camera. These parameters are available due to the preceding scene reconstruction, pose estimation process, and camera characteristics. Depending on the information available, there are two different approaches for calculating the 3D coordinates.

The first strategy requires the availability of depth images containing the depth for each pixel of the respective camera frames. If this depth information is available, the detections’ 3D position can be retrieved based on their bounding box position in the respective frames. This required depth information is available natively for binocular cameras that can capture 3D images [16]. For mono cameras, the depth information can be estimated as described in Section 3.3. This means that in our case, depth maps exist for all videos that were processed in the dynamic scene reconstruction module.

**Figure 10:**
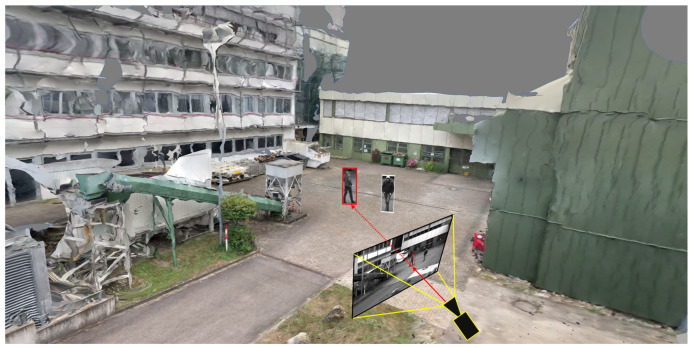
Its extrinsic parameters define the world coordinates of a camera in a 3D scene (camera icon). Based on intrinsic parameters, the pixel coordinate position of an object can be transformed into its respective world position through raycasting. A ray (red line) is emitted through the image at the lower edge of the bounding box of a detection (red rectangle in the camera frame). The intersection of the ray with the mesh provides the related 3D world coordinate.

As depth maps may be noisy and thus lead to faulty 3D localizations of detections, we provide an alternative strategy based on the 3D mesh created in the scene reconstruction step (see Section 3.2). *Raycasting* is applied to calculate the 3D position of a detection. As shown in Figure 10, originating from the estimated camera coordinates (Section 3.2), a ray (red line) is emitted through the bottom center of the detection in the image. The intersection point of the line with the geo-registered mesh is then used as the 3D position of the detection. To determine the angle of the ray, the estimated camera intrinsic properties (focal length and lens distortion) are used in combination with the camera space pixel coordinates of the detection. This approach is based on the assumption that objects must reside at the ground. With objects that are not at the ground, this strategy is, of course, prone to errors. For instance, if a person jumps, then this approach would calculate a wrong position for the time span during the jump, which is farther away from the camera than it is actually the case. To mitigate such inaccuracies in the future, it might be helpful to consider the position and direction of object shadows [66]. Unfortunately, such shadow algorithms depend heavily on lighting conditions. The consideration of shadow effects will therefore probably not eliminate all existing problems of this position extraction task.

Another promising approach builds on the availability of multiple cameras which capture the same detection from different angles. It would be possible to perform a triangulation of emitted rays from several cameras to detect the position of an object in the 3D world space [67]. Overall, the simultaneous application of different positioning strategies allows for modularity by prioritizing more accurate procedures. In the future, this modularity allows to extend the available methods, for example, by camera triangulation and shadow position estimation approaches. Eventually, the proposed prototype enables analysts to view the original footage, which is crucial for confirmatory analysis and critical decision-making processes.

### 3.5. Temporal Footage Synchronization

Another factor why a detailed analysis of video footage is time-consuming and costly is the inaccurate temporal synchronization of several cameras that usually originate from heterogeneous sources. Available video footage must be temporally synchronized so that analysts can get an overview of an incident. This temporal synchronization accuracy also affects the resulting quality of feature extraction methods that require multiple cameras (see Section 3.4). The available footage is often not temporally appropriately synchronized, resulting in poor analysis results, which may even lead to false assumptions that impede accurate decision-making. Therefore, it is crucial to identify the correct temporal synchronization before the information is used in further analysis steps. Even minimal time differences may have a significant impact on critical decision-making processes.

The most basic strategy for performing time synchronization is to use the meta-data of a video to determine its start time. However, the information stored in video files is often incorrect. The system time in cameras may be inaccurate due to manual settings, or the stored creation time is overwritten when the video files are converted or copied. For small adjustments, analysts can manually manipulate the time offsets by to use appropriate values and adjust these offsets for each camera separately. However, this method is tedious, time-consuming, and error-prone, especially as the amount of the video material increases. Therefore, it is advisable to use available auditory or visual features that appear in the video content. For example, analyzing the cameras’ soundtracks to extract distinctive audio features such as shots, shouts, or other significant noises could enable (semi-)automatic temporal synchronization to reduce the manual effort of the analyst. Additionally, it may be helpful to consider the visual features of video frames to identify similarities of events and synchronize the video material based on such anomaly conditions. For instance, the appearance of outstanding visual elements, such as a red bus driving through the scene at a specific time, could be used to match different videos temporally. Moreover, the trajectories of detected objects or persons could be compared and used for temporal synchronization. Enabling analysts to inspect the automatic temporal synchronization is essential for verification. For example, providing a time-aligned list of all videos enables the user to see and compare aligned frames.

So far, the proposed system only supports the first hands-on approach, which is feasible for scenarios with few cameras. As discussed, this approach does not scale for large numbers of videos, which would require the implementation of automatic algorithms that can be monitored by analysts. However, in line with the modular approach of the entire framework, we plan to integrate additional temporal synchronization modules, which can be selected in the preprocessing step depending on the available information.

### 3.6. Preprocessing Run Times

Preprocessing times vary and are highly dependent on the current constellation of modules, their configuration, the input data, and the underlying hardware infrastructure. The proposed pipeline comprises standalone modules that were benchmarked individually by their respective authors. Nevertheless, in the following, we provide a rough overview of preprocessing times for the previously presented constellation of modules on a conventional consumer desktop PC (GeForce GTX 1080Ti, 32 GB RAM, SSD, Intel i7-6700K). The considered data set consists of one handheld camera video (2 min, 1080 p) and three static camera videos from surveillance cameras recording a scene (1.5 min each, 1080 p). For the static 3D scene reconstruction, mainly frames from the moving camera are taken into account (two-minute video). A sparse reconstruction can be created within 10 min. The following dense reconstruction requires approximately 90 min to complete. It is noteworthy that this step only has to be completed once and is not affected by additional static cameras that are embedded in the scene. The given example data set comprises three time-dependent videos which are reconstructed as a dynamic scene. The dynamic object detection and segmentation, as well as the monocular depth estimation, requires about 250 ms per frame. For the given example data set, this results in about 27 min for 6480 frames. This process can be sped up by only reconstructing keyframes. In particular preprocessing times of the high-level analysis are highly dependent on the content of videos, i.e., if many objects appear in the scene, the time increases, and vice versa. Object detection, re-identification, skeleton extraction, and 3D pose calculation require roughly 500 ms per frame. For the given example dataset, this results in an overall run time of 54 min. In the current configuration, the modules were opted for high-quality results. By tweaking parameters, for instance, by disabling multi-resolution object identification in the YOLO module, processing times can be sped up significantly.

In summary, the preprocessing pipeline required approximately 181 min to process the example data set and display the result in an enriched 4D scene, which can be explored interactively. To allow for fast analysis procedures, all individual modules can be tweaked at the price of lowering the quality of results. Additionally, thanks to the modularity of the pipeline and highly parallelizable modules, preprocessing computation can be outsourced to more powerful GPU clusters, shrinking preprocessing times to a fraction of the ones described above.

## 4. Visual Exploration of 4D Reconstruction

**Figure 11:**
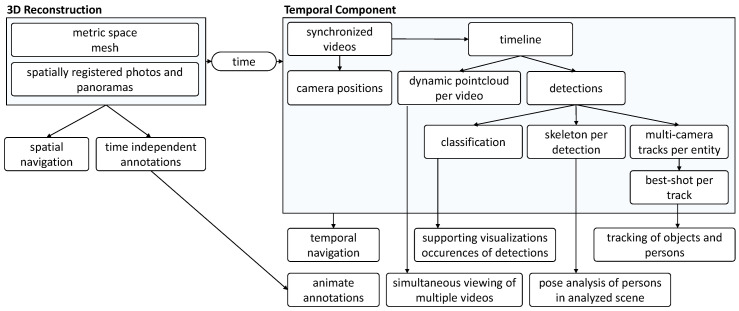
Main elements of the analysis application.

After completing the preprocessing pipeline, analysts can inspect and examine the reconstructed scene using an interactive application. Figure 11 depicts the main building blocks of the analysis application. On the left is the 3D reconstruction, including a static mesh of the given environment and spatially registered time-independent materials such as photos and panoramas. This environment can be spatially explored and enriched with annotations, even if no time-dependent materials were added to the analysis. On the right are all time-dependent materials, such as cameras with estimated locations in space per timestamp, dynamic point clouds, and extracted meta information. We provide a video to demonstrate an exemplary visual exploration of a 4D reconstruction (https://www.youtube.com/watch?v=bcDrLCaI2RI). In the following, most examples are taken from a 4D reconstruction based on the dataset provided by Pollok [68]. The dataset includes several scenes in which several persons, cars, suitcases, etc. are visible and actors reenact different scenarios (e.g., kidnapping and dropping suitcases). It contains video material from three static surveillance cameras as well as footage from handheld devices monitoring the reenacted incident.

**Figure 12:**
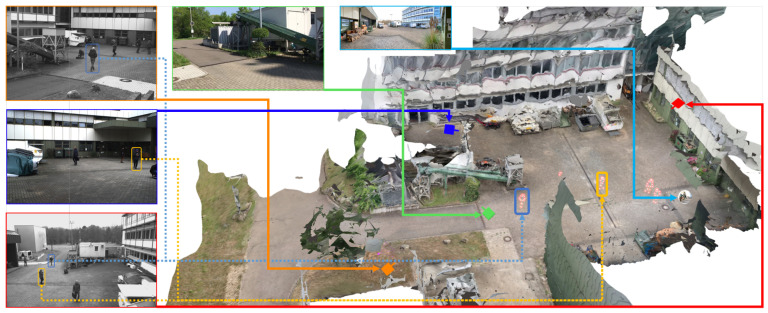
Multiple data sources are bundled and displayed simultaneously in a shared context. On the left side, three frames from static surveillance cameras are displayed. Their locations are indicated by small camera icons in the 3D scene (orange, blue, and red). Detections from all cameras are displayed simultaneously in the scene (dashed lines) as well as static material, such as photos (light green) and panoramic images (teal).

Figure 12 gives a first impression of the benefit of the presented approach. The static 3D reconstruction serves as a base visualization in which all input sources can be placed within a shared context. Static elements such as photos (green) and panoramic images (teal), which provide additional contextual information about the environment, can be spatially registered. Their positions are indicated as camera icons or spheres. Video sources are also spatially registered and visualized as camera icons (orange, blue, and red). This visualization gives the user a good overview of all available input sources and their spatial distribution. It allows the user to relate sources to each other and, for example, find all cameras directed to a certain point of interest. Last but not least, the video footage is temporally synchronized and automatically extracted meta information from all sources can be displayed simultaneously. Blue and yellow dashed lines mark skeletons of detected persons, which were found in different videos. In the following, the building blocks of the demonstrator are described in more detail.

### 4.1. GUI

**Figure 13:**
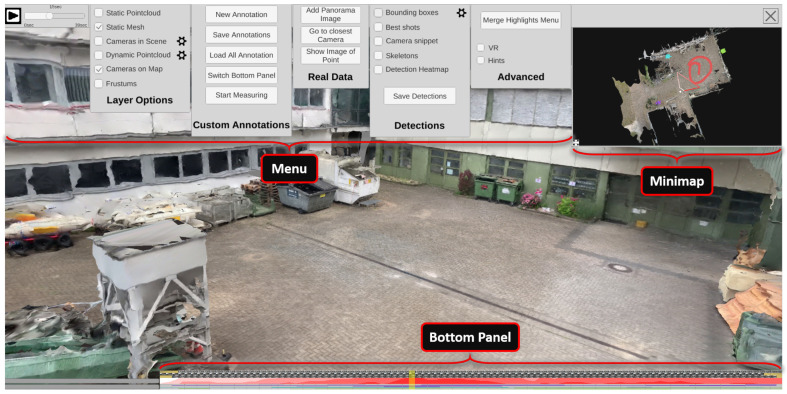
The graphical user interface of the presented demonstrator consists of four main parts: a menu at the top, a minimap at the top right, a bottom panel, and the main window as a view of the inspected scene.

The ISRA-4D interface for the visual exploration of the 4D reconstruction comprises four main parts. As shown in Figure 13, the view of the reconstruction (center) is surrounded by three panels: a menu bar at the top, a mini-map in the top right corner, and a timeline panel at the bottom.

#### 4.1.1. Menu Bar

The menu bar at the top (see Figure 13) allows the user to configure the appearance of the inspected scene and provides options for additional user interactions. A time slider at the top left corner with a play/pause button is followed five menu panels. The first panel (*Layer Options*) allows the user to switch visual layers, such as static and dynamic point clouds, the static mesh, or camera icons in the main scene. The second tab (*Custom Annotations*) provides functions for adding, loading, saving, and changing manual annotations. The third tab (*Real Data*) contains three interaction options for entering or retrieving original photo/video footage into or from the scene. Next to it, there is a panel (*Detections*) for configuring the appearance of automatically extracted content, i.e., detected persons and objects. For instance, users can determine whether bounding boxes should be displayed or skeletons should be drawn into the scene. The last tab (*Advanced*) contains additional functionalities for manually editing and saving automatically extracted detections as well as options for configuring the VR interface.

#### 4.1.2. Minimap

**Figure 14:**
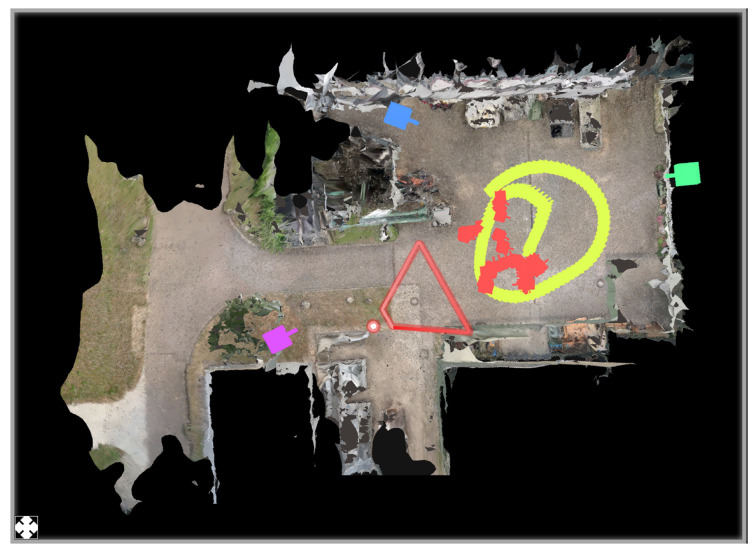
Minimap depicting a top-down view of the reconstructed environment. The locations of the cameras recording the investigated incident are displayed as small camera icons (3 static cameras: blue, green, and magenta; 2 moving cameras: red and yellow). The current location of the user is shown as a small dot, with a red frustum indicating the viewing direction (center) and field of view.

To keep an overview while inspecting the scene, a minimap at the top right provides a birds eye view of the environment (see Figure 14). The current position and viewing direction of the observer is indicated by a red dot and a frustum of pyramid. The minimap’s size can be arbitrarily changed by using drag and drop on the small icon at the bottom left. If cameras are active at the currently selected time, their icons can also be displayed in the minimap, giving the observer an overview of all sources that were monitoring the scene at the selected time. Selecting a camera in the minimap changes the viewport of the main window to that of the selected camera, allowing the user to inspect the scene from the perspective of the source and, if desired, view the original video footage.

#### 4.1.3. Bottom Panel

The bottom panel represents a timeline that covers the period from all time-dependent contents (see Figure 13, bottom). By default, it is collapsed, but it unfolds when the mouse cursor is moved over it, revealing frame previews and additional visualizations about the class distribution and the duration of detections in the scene. See Section 4.4 for more information on the visual elements in the bottom panel. A transparent yellow slider indicates the current temporal position. By clicking on the timeline, the analyst can choose to view a specific point in time manually.

### 4.2. Reconstruction (3D) & Spatial Navigation

**Figure 15:**
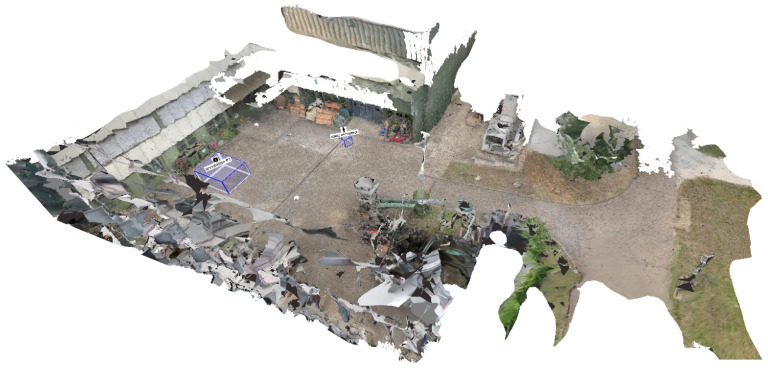
3D scene that can be inspected by flying around in it, which interactively changes the perspective.

Time-independent elements comprise a static 3D reconstruction of the environment, including photos and panoramas that are temporally not registered, and manual annotations (see Figure 11, left). All these elements are static and can be explored independently of temporal navigation. The static 3D reconstruction of the environment serves as a base visualization. Figure 15 shows an exemplary 3D reconstruction. The user can navigate through the scene using standard input modalities (mouse and keyboard). The virtual camera can be moved with the keyboard and rotated with the mouse.

#### 4.2.1. Photospheres and Time-Independent Materials

**Figure 16:**
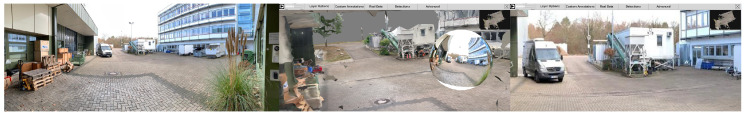
Panoramas (**Left**) are displayed as spheres in the scene (**center**). By opening a sphere, the user “enters” the photosphere to inspect it (**right**).

Additional materials collected for the respective environment can be embedded into the scene, including forensic evidence photos of a crime scene, panoramic shots, and photospheres. These expand the context provided in the analysis, or the analyst can employ them for pre-post comparisons. Figure 16 shows how a panoramic image (left) is displayed within the 3D scene as a textured sphere (center). Once the user clicks on the sphere, the virtual camera is moved to the location of the sphere and the panorama is blended over the 3D reconstruction (right). The user can then “look around” in the 3D scene by similarly using the mouse as before. Photos, photospheres, and panoramas can also be inserted live during the entire analysis process. For example, if the automatic positioning of panoramas is not correct or if new sources became available after the reconstruction was completed. For that to happen, the user can press the corresponding button in the top menu (see Figure 13, “Real Data”), position the new sphere, and select the image from the hard drive to be inserted.

#### 4.2.2. Annotations

**Figure 17:**
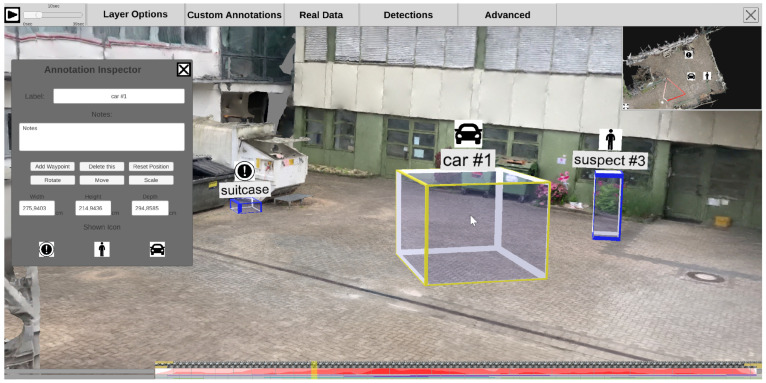
Static user annotations can be manually added to the scene.

Users can further enrich the scene by manually adding static annotations (see Figure 17). The respective interaction for adding a new annotation can be selected in the top menu bar. The user then defines the position of the annotation in two steps. First, a horizontal plane must be selected, which determines the height of the annotation. Second, users can select a position on the plane, allowing them to pick the desired 3D location on the 2D screen. After setting the location, width, height, and depth, additional information of the annotation can be altered. For example, the user can specify a custom logo, enter an annotation label, and type notes.

### 4.3. Dynamic Content

Time-dependent elements include temporally registered cameras (static and moving), dynamic point clouds, detections from video footage, and animated user annotations (see Figure 11, right). All these elements are registered on the global timeline and can be explored by temporal navigation.

#### 4.3.1. Temporal Navigation & Timeline

For easy access, the timeline is displayed twice—at the top left corner and, in large, in the bottom panel (see Figure 13). The currently selected time is indicated by slider bars in the respective timelines and numbers in the upper left-hand corner. The time range is automatically extracted from time-dependent sources as the time span from the global minimum time to the global maximum time. To navigate through time, the user can drag the timeline handles or click anywhere on the timeline. With the play button in the upper left corner, the time can be automatically increased continuously, analogous to the real-time progression. Once clicked, the button turns into a pause button that can be used to stop the automatic increment in time.

#### 4.3.2. Camera Positions

**Figure 18:**
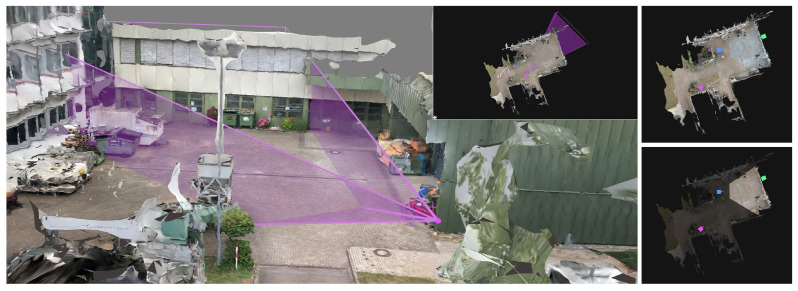
The camera frustums displayed in the scene and minimap can be customized: either as semi-transparent objects (**Left**) or using additive (**top right**) or subtractive (**bottom right**) lighting.

All time-dependent video sources are registered in the timeline, and their location is indicated by a small camera icon that can be made visible if required in the scene and the minimap, separately. Camera icons are only visible if they provide footage for the currently selected time on the time slider. As shown in Figure 18, different types of frustums can be displayed, highlighting the area in the 3D reconstruction that is covered by the video material of the respective camera. On the left side, a semi-transparent frustum is inserted into the scene, clearly showing a cut through the scene where the viewport ends. On the right are two alternative frustum options that work with illumination. At the top right, the camera projects a colored light into the scene that illuminates everything seen by the camera. At the bottom right, a subtractive approach is shown, which hides everything that the camera cannot see.

Camera icons in the scene and on the minimap can be clicked to take a look at the scene from the perspective of the selected camera. Once a camera is selected, it is possible to view its original video material. Additionally, the user can select interactions from the menu bar option “Real Data” to jump to the nearest camera to discover original footage for a potentially interesting perspective. The user can also select a point on the 3D reconstruction to retrieve all cameras with the chosen point within their field of view at the currently selected time on the time slider.

**Figure 19:**
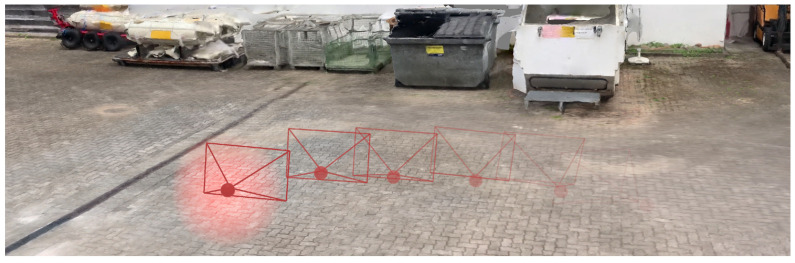
The user can configure the display of moving cameras in the scene. The location of the camera at the currently selected time is highlighted with a red halo. In this example, the camera locations of the last four time steps are also shown with increasing opacity.

Moving cameras change their location in the scene over time. Therefore, a camera icon is created for each frame, as shown in Figure 19. The camera position at the currently selected time is highlighted with a red halo around the respective camera icon. This view helps to get an overview of where a camera has moved to and which areas are generally covered. The user can configure the display of moving camera frustums to reduce clutter. The user can set for how long after and before the currently selected time dynamic frustums should be displayed. This makes it possible to show only one frustum that changes its location over time or to show the current one with any number of preceding and succeeding camera icons. Transparency is used to encode temporal distance.

#### 4.3.3. Detections

**Figure 20:**
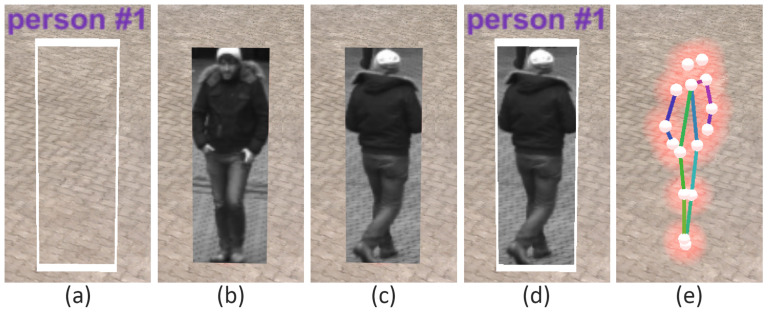
(**a**) A detection can be displayed as a bounding box, (**b**) the best shot of its track, (**c**) the corresponding snippet from its frame, (**d**) a combination of bounding box and best shot or frame snippet, or, if available, (**e**) its skeleton.

Each time-dependent video is preprocessed in a feature extraction pipeline (see Section 3.4). The generated information can be visualized in the 4D reconstruction. Using the top menu bar, the user can configure how detections are displayed. As shown in Figure 20, a detection can be represented as (a) an abstract minimum bounding box with a title; (b) the best shot of its track; (c) the snippet of the minimum bounding box from the original video, as (d) a combination of a, b, and c; or, in case the detection belongs to the class “person”, (e) a skeleton. Detections are displayed with regard to the currently selected time on the time slider. This means that all detections found in different sources at the selected time are displayed. The user can select the sources (cameras) from which detections should be displayed. Additionally, it is possible to filter detections according to their class, so that only detections of certain classes, such as suitcases and bikes, are displayed.

**Figure 21:**
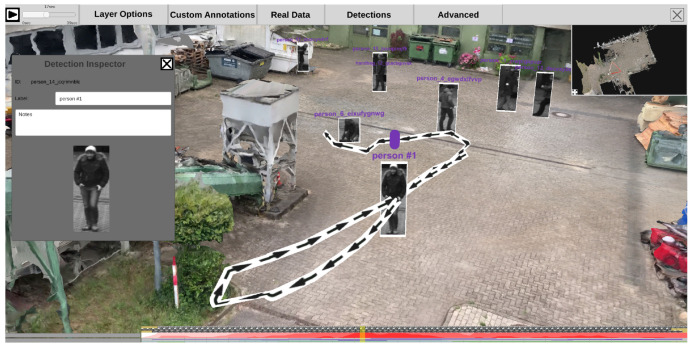
The trajectory of a selected detection is visualized as a directed path within the scene. A menu allows to change the displayed title of a detection and to leave notes.

By clicking on a detection in the scene, a context menu appears on the left side (see Figure 21). This menu allows the user to change its label and add notes. Once a detection has been selected, its track trajectory is visualized in the 3D scene, depicting its spatial progression over time (see Figure 21). Black arrows on the trajectory indicate the direction of movement.

The demonstrator comprises tools to manually refine and adjust automatically extracted information in case the automatic approach did not work as desired. If, for example, two persons who look similar cross in front of a camera, their tracks may get mixed up, and the information displayed is incorrect. The user can solve such issues by splitting the respective tracks and then merging the related tracks.

**Figure 22:**
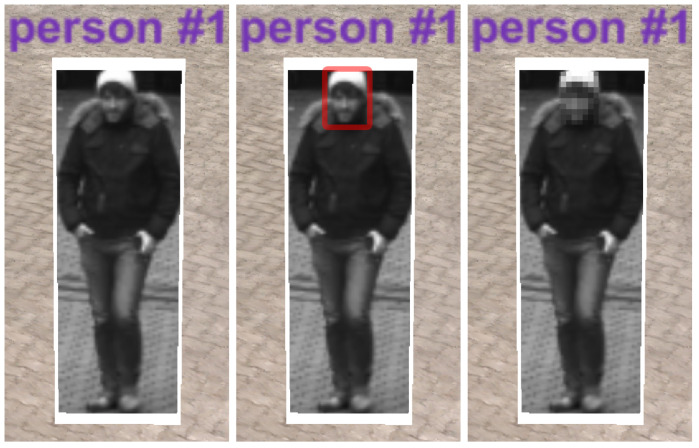
Faces of displayed persons within the 4D reconstruction can be anonymized for privacy reasons. A face detection algorithm detects the bounding boxes of faces (**center**) which are subsequently blurred in the displayed content throughout the visual analysis (**right**).

Identities can be anonymized to protect the privacy of persons in the 4D reconstruction. For instance, if the tool is deployed for an investigation at a public place, faces of bypassers who are not subject to the investigation itself can be pixelated, making them unrecognizable. As displayed in Figure 22, a face detection algorithm detects bounding boxes of faces, and the respective area is pixelated. Throughout the analysis, investigators can reveal the faces of persons that are relevant to the case. A right management system could be deployed to regulate face revelations and define who can use this function.

#### 4.3.4. Dynamic Point Clouds

For each time-dependent camera in the scene, a dynamic point cloud is extracted in the preprocessing step. During the inspection of the scene, the 3D point clouds of all cameras recorded at the currently selected time can be displayed simultaneously. In this way, it is possible to perceive the content of several videos at the same time and in a mutual spatial context without much mental effort. While playing, the observer can fly through the scene and observe the progression of events from different perspectives.

Of course, it is possible to select which cameras are to be displayed as 3D point clouds. The advantage of this technique is that, unlike automatically extracted meta-information (e.g., detections), the original video footage is completely mapped into the 3D scene. Due to detection or classification errors, certain people or objects may not be detected in a frame and, therefore, not be displayed in case the option to show all detections in the scene is selected. However, if the point cloud of the respective frame is visualized in the scene, each pixel of the input frame is also displayed.

**Figure 23:**
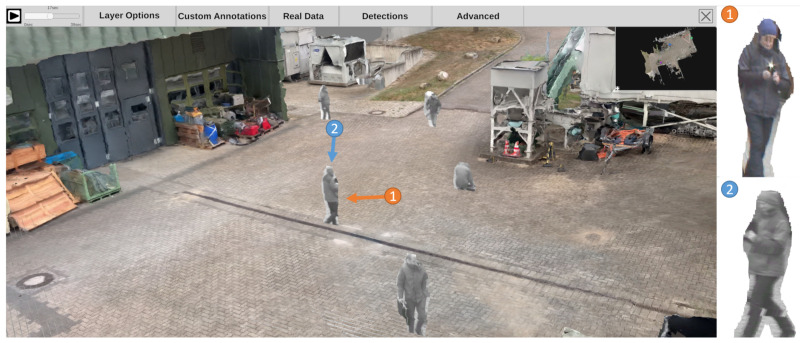
Dynamic point clouds displayed in the static scene from the current perspective (**Left**). If one navigates through space, the perspective changes and point clouds generated from different cameras can be perceived. For example, (1) the (**top right**) point cloud snippet can be seen from the direction indicated by (1) the orange camera and (2) the (**bottom right**) one from the direction indicated by the blue camera.

If the point cloud is viewed from the camera location, it resembles the original video footage. Figure 23 shows an example of the dynamic point cloud visualization in the reconstructed environment. In the current perspective, point clouds are displayed as shown on the left. However, when navigating through space and observing, for instance, a person from different perspectives, different point clouds can be observed from cameras monitoring the scene from respective directions (e.g., (1) from the orange and (2) from the blue viewing angle).

#### 4.3.5. Animated Annotations

**Figure 24:**
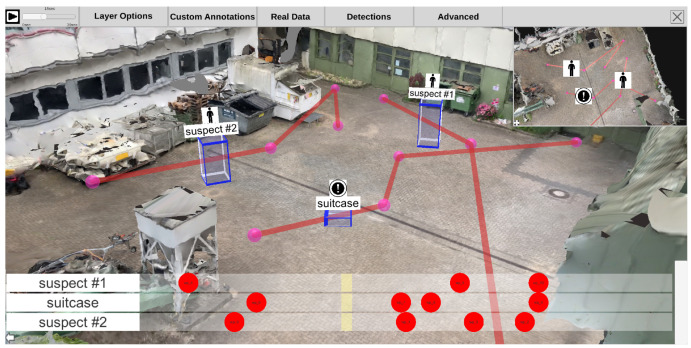
To animate annotations, waypoints can be set and arranged on a timeline that temporarily replaces the bottom panel. Waypoints determine the location of an annotation at a particular time.

Static annotations can be animated and thus integrated into the global timeline along with videos and detections. To do this, the user can select the annotation to animate and select the option to add a waypoint. This action creates a waypoint for the annotation’s current location, and another can be set interactively, similar to the location selection of annotations (see Section 4.2). When adding waypoints, the bottom panel view is automatically changed to a waypoint timeline view (see Figure 24). Each annotation is displayed in a list, and all corresponding waypoints are lined up for each annotation. The user can shift waypoints in time to define the location of the annotation at a specific time. If two waypoints are at different locations, the annotation position is interpolated depending on the time between the two waypoints. In this way, the user can, for example, reconstruct a course of events described by eyewitnesses or plan the progression of an intervention.

### 4.4. Visual Analysis

**Figure 25:**
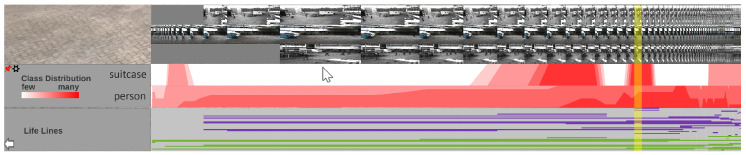
The bottom panel consists of three elements: At the top is a frame preview of all selected cameras. In the center, the class distributions of the detections are visualized as horizon charts. At the bottom is a chart depicting the appearances of all detections as lines.

The bottom panel can be expanded by hovering over it with the mouse, as depicted in Figure 25. Optionally, it can be pinned to stay open and reveal the underlying visualizations while navigating through the environment. When it is enlarged, three visualization elements appear. At the top, all time-dependent video frames are aligned in a scrollable and filterable list that indicates the start and endpoint of each video on the global timeline and provides a first glance at the original video footage. The visibility of the frames is supported by a fisheye effect induced by hovering over the frames. Dependent visualizations accordingly scale while hovering.

The other two elements (center, bottom) are visualizations of automatically extracted detections. A horizon chart visualization shows the distribution of a particular class of detections over the entire time axis in the center. The user can select classes and combinations thereof to be displayed as individual horizon charts. For each selection, a horizon chart is displayed, showing the total number of detections of the selected class(es) at each time. In the example (see Figure 25), one horizon chart was created for all detections of the “suitcase” class and another for the “person” class. With the given visualization, it is easy to identify at what times a particular object type was detected to jump to it quickly. Besides, this visualization allows the user to identify periods when nothing was detected, which in surveillance use cases, for example, helps to sight large amounts of video material more quickly.

The last visualization element (bottom) shows the time spans of detected tracks. Each row belongs to a single object and is filled with color when it was detected in the original footage. The line’s color represents a visual link to the corresponding video in which the track was detected (colored camera icons in scene and minimap). The sources of the detections visualized in the bottom panel can be filtered as desired to analyze only one video or a subset of videos.

**Figure 26:**
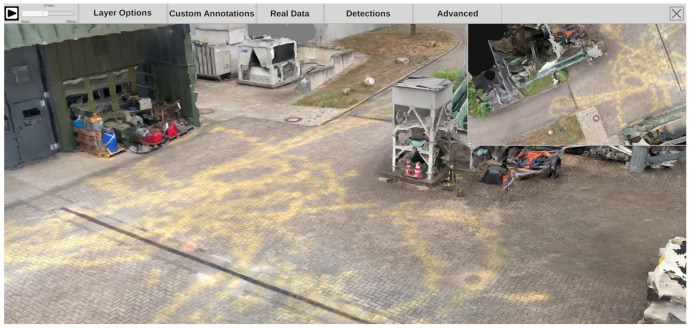
A heatmap visualization of selected detections can be projected onto the environment providing an overview of where objects or persons were detected in the analyzed scene.

Additionally, the current selection of detections (after filtering by classes and video sources) can be displayed as a heatmap projected onto the reconstruction (see Figure 26). Once the user selects the respective option in the top menu bar (Figure 13, “Detections”), a 2D heatmap with the locations of all selected detections is created in the background. A grid of 10×10 cm tiles with the environment’s size is used to create the heatmap. Each tile counts how many detections are in its corresponding area. The resulting n×m grid is smoothed with a Gaussian kernel, normalized, and finally saved as an image in which the corresponding tile value determines each pixel on a user-defined color gradient (from transparent to yellow to red). The generated image is then projected orthogonally onto the mesh.

With the heatmap visualization, the user can quickly identify which areas of the environment contain most occurrences of persons and objects and which areas were uneventful. For example, by filtering for all detections of the class “suitcase” and displaying the heatmap for it, the user can quickly see where a suitcase was detected to find all video sources which monitored the respective areas. Especially in combination with horizon chart visualizations, it is easy to find all videos in which and the corresponding times at which suitcases were spotted.

**Figure 27:**
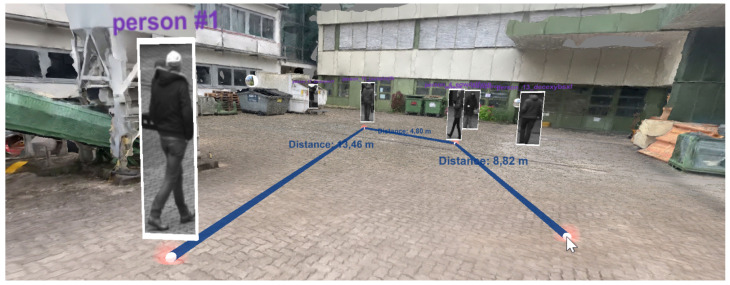
Interactive tool for measuring distances and object sizes in the reconstruction.

Throughout the analysis, the user can insert images or spatial and time-dependent annotations. Additionally, the user can activate a measuring tool in the top menu (Figure 13, “Measure Distance”). Once activated, measuring points can be created by clicking on the environment (see Figure 27). Intermediate segments are labeled with their distance in meters, and the total length is displayed in the top menu.

### 4.5. VR Exploration

**Figure 28:**
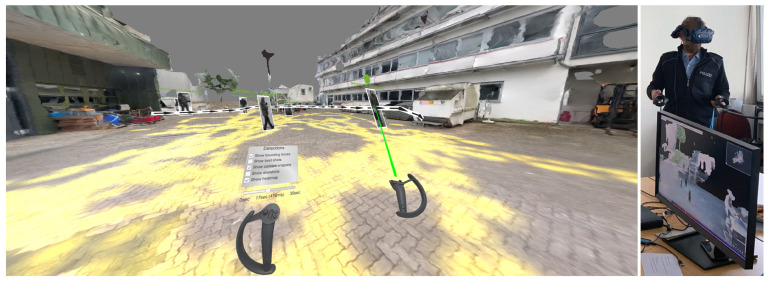
(**Left**) View of an exemplary scene in VR. (**Right**) Set-up with immersed investigator.

Besides exploring the 4D reconstruction on a monitor screen, it is possible to enter the scene in virtual reality (“VR”). When a VR headset is connected to the PC, it is automatically recognized and configured for usage. In the current example, we use a Valve Index VR head-mounted display [69]. The environment is scaled to metric space, which means that the environment is represented as a life-size model. Distances and dimensions of objects can be viewed as in the real world. Figure 28 shows an example scene as it can be observed in VR. All visualization elements like detections, heatmaps, camera positions, and annotations can also be inspected in VR. The user can navigate through space by walking (if the available physical space allows it) or virtual teleportation. The user can press and hold the touchpad on the right controller to select a target location to teleport. When released, the user’s location is set to the respective position.

**Figure 29:**
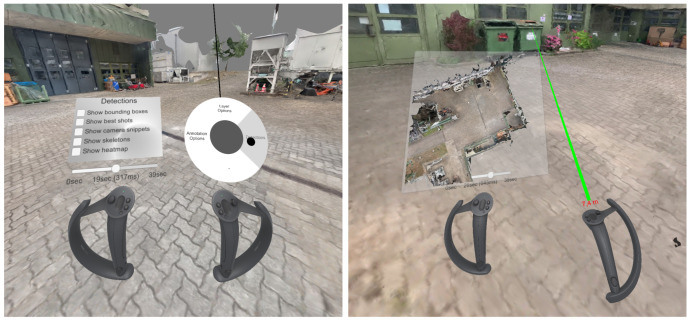
(**Left**) A radial menu can be opened on the right controller to open various menus that are displayed on the left controller to configure the visualized scene. (**Right**) A minimap and a distance measuring tool can be activated on demand.

For further interactions, the user can open a menu by placing the thumb on the right joystick (see Figure 29, left). The joystick can be moved around and released at the desired option to select an item on the radial menu. The selected menu opens and is attached to the left controller. Options can then be selected by pointing the laser on the right controller at them and pressing the right trigger button. In this way, it is possible to modify the appearance of the scene (“Layer Options”), the display of detections (“Detections”), and the annotation of the scene (“Annotation Options”). The user can either drag the slider attached to the left controller or use the left joystick to scroll forward and backward to navigate in time.

A minimap of the environment can be toggled using the “B” button on the left controller (Figure 29, right). The user can click in the minimap to teleport to the selected location. Furthermore, the “B” button on the right controller can be used to toggle a measuring tool. After activation, the laser emitted from the right controller measures the distance to the surface that it hits and displays it above the controller.

Similar to interaction possibilities on the screen, the user can select detections and annotations in VR by pointing at them and pressing the trigger button. When selected, the respective menu is displayed and attached to the left controller. As traditional text input in VR is quite cumbersome, a speech-to-text module allows to change labels or add textual notes to annotations and detections via voice commands. For this, the user can select an input field, hold down the left trigger button, and record spoken input. Afterward, it is converted to text and inserted into the chosen input field.

**Figure 30:**
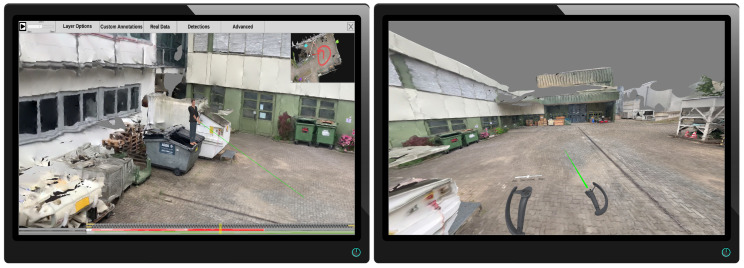
Collaborative setup with multiple monitors connected to the system. One monitor shows the usual view of the 4D reconstruction (**Left**) and the other one, a view from the simultaneous observer’s perspective in VR (**right**). An avatar of the VR observer is displayed in the desktop interface (**Left**).

Our exploration tool allows the simultaneous usage on a screen and in VR. While one collaborator observes the scene in VR, the other can interact as usual on the monitor while seeing the VR user as an avatar walking through the scene (see Figure 30, left). If two monitors are connected to the PC, one depicts the scene’s regular interface and the other the observer’s view in VR (Figure 30). The VR user can activate a laser pointer on the right controller by pressing the right trigger button to point at something for improved communication.

In a initial qualitative assessment, law enforcement officers who had the opportunity to test our demonstrator provided feedback. Eleven criminal investigators of the German Federal Police (Bundespolizei) evaluated the presented demonstrator. Overall, they were convinced of the added value of virtual reality in the given context. The main argument was that it was advantageous to inspect the scene “from within”, as one could perceive the environment in its natural size and explore it by walking around in it. This made it easier for them to estimate distances between detected entities and get a better spatial sense of the scenario. Another potential benefit mentioned was that such virtual tours in VR could be used to inspect crime scenes from a distance without visiting their actual physical location, or to use them at court to illustrate a sequence of events in a criminal incident graphically.

## 5. Use Cases

In the following, four use cases demonstrate the potential of the proposed approach.

### 5.1. Mass Data Analysis & Preparation of Evidence

After major criminal incidents, such as terrorist attacks in a city, law enforcement agencies collect large amounts of evidence. These usually consist, among other things, of surveillance videos and recordings of eyewitnesses (photos and videos). In the case of unusually large incidents, law enforcement agencies even tend to set up platforms for the civil population to upload witness photos and video recordings. In total, the amount of digital information collected can easily exceed thousands of gigabytes of data and months of non-stop video recording. In practice, the review of evidence materials is still primarily done by hand. Criminal investigators go through all photos and videos, assess their relevance, and try to relate them to other materials, e.g., by annotating time, location, and meta information.

However, to cope with such an amount of data and facilitate the preparation of evidence for use at court, automatic mechanisms could be used. Our demonstrator could filter spatially and temporally relevant sources, place them in a shared context, and extract valuable meta information. To achieve this, the region of interest would first have to be specified and reconstructed as a 3D model. To return to the example of a terrorist attack in a city, videos of all affected areas could be employed to create a reconstruction. This can either be videos and photos from the time after the incident (filming the area with handheld cameras or drones) or video footage from the incident, which can be assigned with certainty to the area of interest (e.g., surveillance cameras with known locations). Subsequently, the entire pool of videos and images of the incident is fed into the reconstruction pipeline, trying to find matching points with the original reconstruction. Materials that do not overlap with the reconstruction are filtered out and may be subject to manual inspection. However, for all videos and images that can be registered at the site of the event, their location can be estimated and positioned in the reconstruction. After a semiautomatic temporal synchronization step, footage from before or after the incident can also be filtered out. All remaining evidence can be aligned on a shared time axis.

The 4D reconstruction can then be inspected, providing an overview of the environment, all available video and photo sources and their locations, and the time during which each location was monitored. This approach could drastically reduce the materials that need to be viewed manually. In addition, the approach facilitates the process of bringing recordings of the same area from different angles into a mutual spatial context without much mental effort. Moreover, the automatic approach could assist investigators in the analysis of meta information. For example, it would be possible to track an object or person through a single video or complete footage. In the graphical exploration, an entity’s spatial progression would be displayed as a single continuous path, regardless of the recording’s source.

Besides providing a quick first overview of the evidence, it can be used as a starting point for further adjustments, such as the manual insertion of footage that could not automatically be registered correctly. When all relevant information is in one place and can be located precisely in space and time, it is much easier to keep track of large amounts of evidence.

### 5.2. Crime Scene Investigation

Crime scenes, such as a murder scene, are carefully documented during criminal investigations. After the incident, the police collects forensic footage to record evidence and store as much information as possible about what the location looked like shortly after the crime was discovered. If available, information at the time of the incident is also considered, such as nearby surveillance cameras, witness reports, or even videos and witnesses’ photos.

A 3D model of the crime scene can be reconstructed with the presented demonstrator, showing the crime scene as it was found before clean-up. All available sources are registered in the reconstruction. In this way, the highly unstructured mass of digital information is spatially organized, so that, for example, all sources recording a certain point in space can be easily identified. The 3D model could help criminal investigators organize the available information and put it into a spatial context without much mental effort. It also allows remote inspecting of the crime scene without physical presence and at a later time. For example, as pointed out by interviewed criminal investigators, the reconstruction could be used as an interactive, graphic basis for conveying information at court hearings.

Besides providing a permanent image of the crime scene, footage of the incident itself, if available, can be interlaced. Similar to the previous use case, footage from surveillance cameras and eyewitnesses can be displayed in the reconstruction. Meta information such as person and object detections and their tracks could be automatically extracted and displayed. As the forensic material provides much information about the scene after the incident, pre–post comparisons with the footage could easily be made. For example, suppose a specific point of interest was recorded with a surveillance camera. In that case, this point can be selected in the 3D scene to reveal all original footage sources which contain the same location.

The 3D reconstruction could also be incredibly helpful in reconstructing the sequence of events that led to the crime and a possible later course of events. If, for example, witness reports are available, criminal investigators can resort to animated annotations and try to visually model the described occurrences—possibly together with the witnesses themselves. Having a graphic representation of the environment in front of them could help them remember the course of events more accurately.

### 5.3. Real-Time Surveillance Scenario

**Figure 31:**
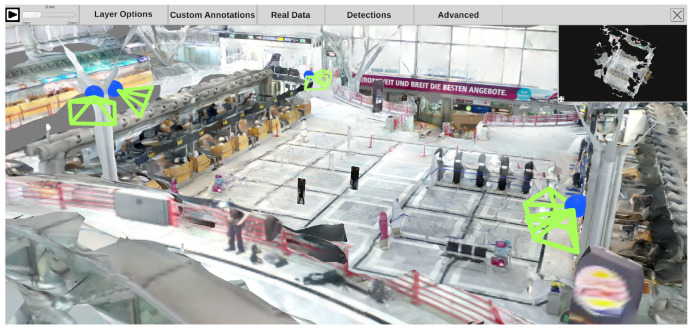
Reconstruction of airport in which multiple surveillance cameras are spatially registered. Video streams of the cameras are fed into the system and automatically extracted detections are depicted in the 3D reconstruction in real-time.

The approach presented could also be used for real-time surveillance tasks. A monitored complex, such as an airport site, can be reconstructed as a 3D model—either with the previously presented method using large amounts of images or with alternative approaches, such as native 3D modeling (e.g., with a floor plan of the building), or 3D laser scanning. All available surveillance cameras are then spatially registered within the model, and their video streams are fed into the system (see Figure 31). Their video streams are processed in real-time in a pipeline for object and person detection. The extracted meta information is continuously displayed within the 3D scene. Security staff can then interactively monitor a single representation of the entire complex, rather than a wall of monitors, showing the footage from a single surveillance camera. Besides, the model would provide a good overview of the distribution of cameras in the complex, making it easier to follow suspicious persons walking past different cameras, even if the cameras’ original video recording is monitored. Especially in use cases like this one, however, it must be critically reflected from a data protection and ethical point of view to what extent technical possibilities should be employed. For example, the tracing and re-identification of a person could already constitute a massive encroachment on a person’s rights.

### 5.4. Mission Planning and Training

**Figure 32:**
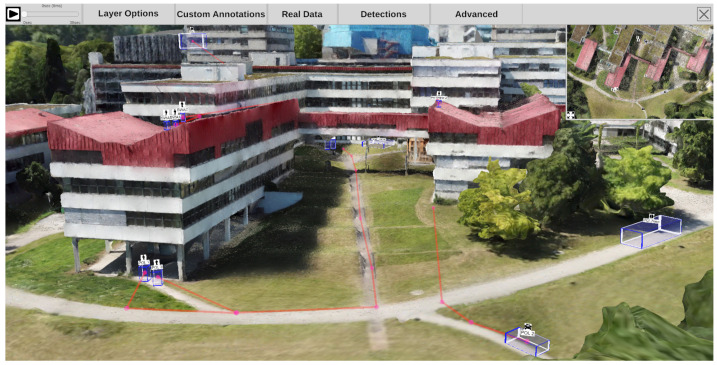
Exemplary reconstruction of the environment for strategy planning in police operations. The demonstrator creates a static mesh from drone recordings. The planned movement of police forces can be sketched in it.

Another use case for the presented demonstrator would be applying 3D reconstructions for mission planning and training scenarios. For example, in the case of a hostage situation in a university, the available video and photo footage of its complex could be used to create a reconstruction. Additional material collected during recon missions by robots or drones can be used to improve the 3D model. Special police forces could then use this reconstruction to get a picture of the surroundings and plan strategies. For example, animated annotations can be used to sketch possible ways to enter the building and free the hostages (see Figure 32).

Furthermore, police forces could use models of past missions or create new 3D models of training environments to train their personal. Employing VR could be of particular benefit in such training scenarios, as trainees can enter the given surrounding and perceive them more realistically. Previous research points to advantages of virtual reality in terms of memorability [49], spatial navigation [70], orientation [71], learning performance on 3D models [41], and the understanding of complex geometries [44], which could also lead to an overall better training effect in the given context.

## 6. Discussion

### 6.1. Limitations

The current version of the demonstrator has different limitations. Like other state-of-the-art 3D scene reconstruction algorithms, the current algorithm is sensitive to low-quality input material. For example, differences in illumination in images taken from the same location may mean that no commonalities may be found between the two sources, leading to the inability to identify spatial links. The image quality is also of great importance. Artifacts caused by motion blur in videos or low image quality, e.g., due to poor illumination during night shots, have a significant impact on the reconstruction quality and can make it impossible to register cameras. Therefore, it is not guaranteed that all videos that record a specific location are also registered in space and thus revealed in the visual analysis process. Additionally, cameras may be incorrectly registered due to a confusion of crucial points. Both cases pose a threat to a possible decision-making process during visual analysis. Therefore, it is essential to verify automatically calculated and extracted information and consult the original data for the final decision making. Although the current framework provides links to original data, the convenience provided may discourage additional verification steps.

In the current approach, the reconstruction can be recognized as a non-realistic estimation of the environment. However, with improving reconstruction algorithms, the environment’s quality and level of realism might align more and more with real-world experiences. Like this, errors in the reconstruction might be accepted without further verification. Therefore, the currently already provided functionalities to quickly open original video and image footage will be important in the future.

We presented our tool’s functionality for navigation through a 4D reconstruction in VR. Although this offers several advantages, it also has drawbacks. Some users are prone to simulator sickness and become nauseous after a short time of immersion, limiting the target group of potential end users. Moreover, even though the illusory reality looks spacious, the physical interaction space is usually limited, resulting in a small area where users can actually walk naturally. Movement-compensating techniques such as virtual teleportation must be used to cover greater distances in VR, but can negatively influence the perceived presence and orientation [71].

Beyond that, the demonstrator presented is intended to convey the underlying concepts and not represent a ready-to-use prototype. Therefore, it cannot currently scale to hundreds of input videos. The preprocessing should be outsourced to a GPU cluster, enabling highly parallel processing to process more massive amounts of input data. This would lead to an almost linear reduction in computation time since most high computing power steps can indeed be parallelized.

### 6.2. Ethical Considerations and Legal Aspects

Naudts and Vogiatzoglou state in “The VICTORIA Ethical and Legal Management Toolkit” that every application of new technology should consider several general ethical principles [72]. The proposed approach requires readily available imaging data. Of course, this data needs to be gathered in a lawful and ethical way. For example, this could include locations such as airports, train stations, or other public places where CCTV cameras are already deployed. To create a proper reconstruction, additional imaging material from a moving camera sensor is necessary. It is best to achieve a high-quality reconstruction if no persons or other moving objects obstruct the view. Thus, no personal data is required for the reconstruction.

The following list of general ethical principles [72] need to be discussed that concern the proposed demonstrator. Beneficence is a principle stating that new technology should improve the individual and collective well-being. Our approach is designed to improve the way users can access 4D imaging data from multiple camera sensors in a more intuitive manner. In general, this measurement can improve the overview of complex scenes, such as an airport, enabling security personnel to detect important events such as an imminent threat and eventually respond faster. In the case of a crime scene reconstruction, a 4D scene may enable criminal investigators and legal experts to improve the decision-making, and the trial process as the specific spatial properties of a crime scene can be investigated exploratory and immersively using virtual reality. This is also relevant in the principle of the right to a fair trial. The same argument is also valid for our use case of mission planning and training, potentially saving lives.

On the contrary, the principle of non-maleficence states that new technology may not be exploited to harm human beings. Our most significant concern here is that our approach may lead to an increase in surveillance, as it allows humans to maintain the overview of a scene even if more camera sensors are being added. On the other hand, deploying CCTV cameras must be aligned to the law and is heavily regulated in many countries.

Justice and fairness are heavily discussed topics in scientific communities [73] and politics [74]. This topic is related to non-discriminatory AI. Both topics are notably complex, and no general solution seems to be available. Naudts describes how this is also reflected in regulations as they are complex and multilayered [75]. The modular system employed in the presented demonstrator also includes the detection of objects and persons. The demonstrator merely receives a class, bounding box, and the respective frame plus additional metadata such as uncertainty values. Our tool displays all available data and does not filter, for example, by uncertainty values to mitigate the problem of fairness. However, the problem that certain aspects of a scene may remain undetected persists, but it is more unlikely the more cameras and frames are available containing the object. Another optional module is the re-identification if this is enabled, it allows the user to track objects through the scene; for example, by visualizing the paths and lifelines. Such modules may require the use of biometric data such as detecting persons by their faces. Therefore, the lawful applicability must be ensured. However, as Kindt argues, clearer rules regarding the use of biometric data are required [76]. This is also heavily affected by GDPR. Our approach is robust to deal with, for example, with blurred faces. The data must be prepared before it is loaded into our tool.

The principle of autonomy states that humans must remain in control over important decisions affecting themselves and others. In the research field of visual analytics, this is also known as the human being, the ultimate decision-maker. In a criminal investigation, interactions with a tool relevant for decision-making must be tracked and presented at court in combination with the findings [38]. We envision our approach and tool as an alternative view for 4D scenes. It does not automatically make decisions except for the reconstruction itself. The tool furthermore always allows the operator to access and view the original data and relevant metadata. This measurement also complies with the principle of explicability and eventually increases the operator’s trust in the system.

### 6.3. Future Work

New techniques and approaches are continually being developed, which are improvements of current steps in our preprocessing pipeline. For example, new, faster, and more accurate ways are developed to detect objects and persons in videos, re-identify them in other frames or videos, and extract metadata from them. The presented demonstrator is based on a modular design that allows the continuous adaptation to technological progress.

In the future, outsourcing the preprocessing pipeline to a GPU cluster should increase processing speed and facilitate the analysis of large amounts of videos. We also plan to improve various steps in the preprocessing procedure, such as the extraction of meta information. Although skeletal data can be easily extracted from detected persons, they are not yet classified for further analysis. Another next step would be to apply behavior classification networks to the skeletal data. As a result, the skeletons would be labeled with tags describing their current state within a particular frame, such as “walking” or “sitting”. We also plan to improve the inter-video re-identification of detections to calculate 3D locations of detections, merge 3D point clouds of the same detection, and create 3D avatars for detected persons.

Currently, the reconstruction of the static 3D environment and the cameras’ spatial localization are computed in a single step. It would be advantageous if sources could be added incrementally to an existing 3D reconstruction. In this way it might even be possible to register a moving camera on-the-fly in the static reconstruction and enable image-based position tracking.

Furthermore, we plan to extend the applicability of the tools for collaborative investigations. Currently, only one person at a time can enter the virtual environment. In the future, however, the remote collaboration of several users in virtual reality should be supported. The option to enter the same virtual environment and explore the 4D reconstruction interactively could improve the dialogue between remotely located participants due to the improved communication basis [77,78]. Future research should assess the possible benefits of remotely co-located collaboration in this context.

In addition to a quantitative evaluation of immersive analytics for the interactive analysis of 4D scene reconstructions, we also plan to systematically evaluate the overall system by assessing its performance for various analysis tasks and comparing it with alternative approaches. For selected databases, users will try to solve specific tasks and extract high-level information from the data. Tasks could range from basic questions such as “How many different people are visible in all videos?” or “Who drops a suitcase when and where?” to more complex analysis tasks such as “Find the person who threw a bottle, trace back where he/she came from, and extract a frontal image of the person’s face”. Besides performance and task completion times, additional measures such as usability and workload will be taken into account.

## 7. Conclusions

This work introduces a framework for the interactive, visual analysis of mass image and video data. The framework consists of a modular preprocessing pipeline that prepares a highly unstructured and heterogeneous bulk of digital footage for later display. Besides the temporal and spatial registration of the sources in a static 3D reconstruction of the corresponding environment, meta-information is extracted for each video. Therefore, the user can spatially and temporally explore the data while maintaining an overview of all materials. The main advantage of this approach is that all information is presented in a shared visual context, which reduces users’ mental effort to link different sources. Besides, the framework enables immersive exploration of the data space in VR, allowing the analyst to “enter” the 4D reconstruction and search it more naturally. To illustrate the versatile applicability of the framework, four use cases for different application areas such as crime scene investigation, real-time surveillance, mission planing, and training scenarios were presented. Initial qualitative assessments by criminal investigators underline the potential of using virtual reality for the exploration of 4D reconstructions, as it fosters spatial understanding, allows more intuitive ways of collaboration, and enables remote inspection of crime scenes in a natural way.
